# Deep learning approaches for natural product discovery from plant endophytic microbiomes

**DOI:** 10.1186/s40793-021-00375-0

**Published:** 2021-03-18

**Authors:** Shiva Abdollahi Aghdam, Amanda May Vivian Brown

**Affiliations:** Department of Biological Sciences, Texas Tech University, 2901 Main St, Lubbock, TX 79409 USA

**Keywords:** Endophytic fungi, Deep learning, Secondary metabolites, Natural product, Endohyphal bacteria, Mycovirus, miRNA, Multi-omics

## Abstract

Plant microbiomes are not only diverse, but also appear to host a vast pool of secondary metabolites holding great promise for bioactive natural products and drug discovery. Yet, most microbes within plants appear to be uncultivable, and for those that can be cultivated, their metabolic potential lies largely hidden through regulatory silencing of biosynthetic genes. The recent explosion of powerful interdisciplinary approaches, including multi-omics methods to address multi-trophic interactions and artificial intelligence-based computational approaches to infer distribution of function, together present a paradigm shift in high-throughput approaches to natural product discovery from plant-associated microbes. Arguably, the key to characterizing and harnessing this biochemical capacity depends on a novel, systematic approach to characterize the triggers that turn on secondary metabolite biosynthesis through molecular or genetic signals from the host plant, members of the rich ‘in planta’ community, or from the environment. This review explores breakthrough approaches for natural product discovery from plant microbiomes, emphasizing the promise of deep learning as a tool for endophyte bioprospecting, endophyte biochemical novelty prediction, and endophyte regulatory control. It concludes with a proposed pipeline to harness global databases (genomic, metabolomic, regulomic, and chemical) to uncover and unsilence desirable natural products.

**Supplementary Information:**

The online version contains supplementary material available at 10.1186/s40793-021-00375-0.

## Introduction

Microbiomes including communities of fungi and bacteria living asymptomatically within plant tissues, are ubiquitous and important components of plants. Specialized microbes within plants harbor capacities to synthesize diverse and unique secondary metabolites (SMs), hence, they have been a major focus for anticancer, antibacterial, antifungal, and antiviral natural product (NP) discovery [1–6]. Even though most plant microbiome species are exceedingly challenging to work with, being difficult to grow and unlikely to express most SMs in culture, interest in them as a source of medically important NPs has exploded, catapulted by the discovery of the breakthrough anticancer compound paclitaxel (Taxol) synthesized by the endophyte *Taxomyces andreanae* from Pacific yew trees (*Taxus brevifolia*) [7–10]. Research since the discovery of paclitaxel shows plant microbiomes, particularly the internal endophyte communities, offer a treasure trove of bioactive secondary metabolites with at least 60% of characterized species having medical and drug potential due to their novel and novel chemical structures [4, 11–13].

Familiar endophyte-derived medically important compounds include anti-cancer drugs paclitaxel, comptothecin, vinblastine, anti-viral drugs podophyllotoxin, isoindolone, talaromyolide, cytonic acid, and anti-bacterial drugs altersolanol, cryptocandin, and rutin [4, 14–18]. Indeed, microbes, rather than plants, dominate the pool of identified sources for drugs, representing about 75% of candidate drug sources, generating between 15 and 30 approved new drugs per year in the U.S. with indications for over 70 conditions or diseases [19]. It has been argued that plant microbiomes present a vast underexplored resource for discovery of chemically diverse NPs that may rival that from free-living microbes [20]. This phenomenal potential could be due to their ~ 400 million years of intimate service to plants [21, 22] in which endophytes evolved in a context of exceptional biochemical demands [23–27] leading to novel SM synthesis.

Whereas the majority of SMs exist in apparently silent gene clusters [28–31], if unsilenced, we estimate that global plant microbiomes may potentially yield 1.3 to 28.3 × 10^9^ NPs that could lead to millions of drugs (see calculations in Tables 1 and 2). This biosynthesis needs only to be awakened – analogous to waking the sleeping giant – but so far, the path forward to harness this potential has been unclear. Significant barriers exist that prevent progress in endophyte NP discovery. For example, genome sequencing and bioinformatics predict a vast pool of compounds missing in culture-based studies [47, 51, 55, 57–59] that fail to be expressed except *in planta*, or without providing substrates or precursors from plants or other microbes [28, 60]. Regulatory breakdowns that limit endophyte NP expression include spatially and temporally varying signals from the plant, other endophytic fungi, other endophytic bacteria, endohyphal bacteria [61–67], and perhaps even phage or mycoviruses [68–70]. There is also evidence for cooperative synthesis of compounds predicted in the hologenome [61, 71, 72].

**Table 1:** Estimating plant microbiome diversity and NP potential on Earth

**Fig. 1:**
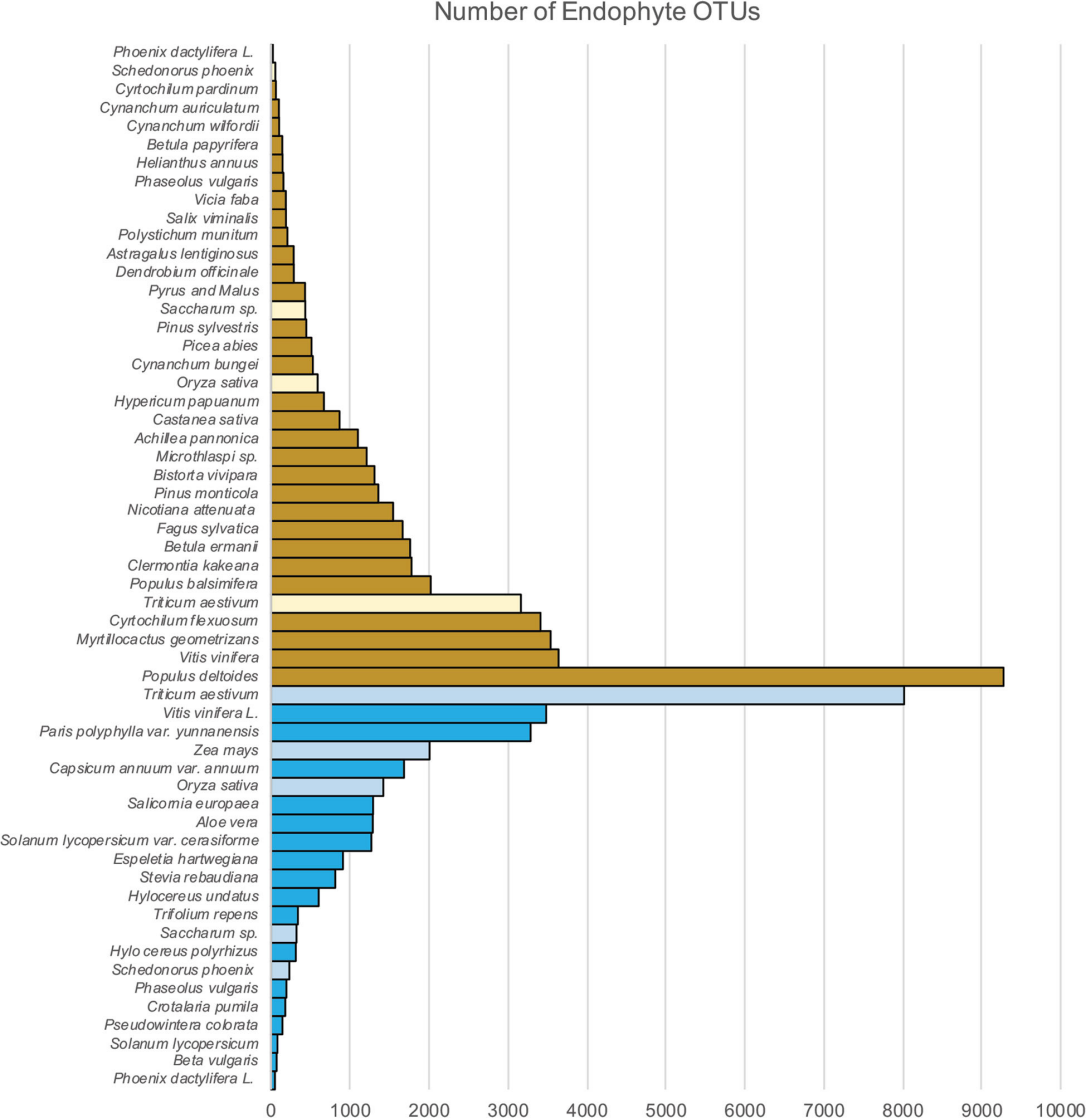
Endophyte richness in OTUs per plant species, based on cultivation-free amplicon sequencing: ITS or 18S rRNA for fungal endophytes (brown); 16S rRNA for bacterial endophytes (blue); with light shading for species within the grasses (Family Poaceae). Data was compiled from references in Supplementary Table 1

Here, we use simple, additive, non-combinatoric approaches to estimate endophyte species richness. In these calculations, only endophytic fungi and bacteria and considered with the simplifying assumption that each endophyte species acts alone (without the plant or other microbes) to synthesize its NPs ***Historical estimates***: The most widely cited estimate of endophyte species on Earth was proposed over 25 years ago, predicting 1.3 million endophytic fungi on Earth [11]. The simple calculation considered only culturable fungi from vascular plants and was based on researchers’ experience suggesting each plant species hosts 2 to 5 unique host-specific endophytes (thus, for 270,000 plant species there would be up to 5 × 270,000 unique endophytes). While this study did not estimate global NPs, the following calculation attempts to do this. This study argued that each phylogenetic cluster of fungi produced largely similar set of known secondary metabolites which largely differed from that of other clusters. For the 8 well-studied groups of endophytic fungi in [11], comprising 8300 species there are an average of 1038 species per group, which would comprise 1253 groups (1.3 million/1083 species). Together these groups reportedly produced 5351 unique known secondary metabolites, or an average of 669 secondary metabolites per group, which for 1253 groups would produce an estimated 838,100 unique secondary metabolites on Earth. Among the shortcomings of these estimates are omission of bacterial endophytes and non-culturable endophytes, and omission of novel metabolites and predictions of silent or cryptic biosynthetic clusters. ***Estimates based on next-generation sequencing****:* Based on amplicon sequencing of endophytes from plant tissues using 16S rRNA and ITS or 18S rRNA genes revealing large numbers of previously uncultured endophytes (i.e. OTU-based surveys, Fig. 1), a simple “back-of-the-napkin” estimate suggests there may be at least one non-culturable host-specific fungal endophyte for every one that is culturable [23], and perhaps 10 host-specific bacterial endophytes, such that for the estimated ~ 300,000 plant species on Earth, there may be 10 fungi + 10 bacteria (=20) × 300,000 = 120 million endophyte species on Earth. Whereas this is two orders of magnitude greater than historical estimates, this would constitute only 0.012% of the estimated 1 trillion microbial species on Earth [32], suggesting it is not absurdly high. Based on estimates of known metabolites discussed above, this suggests endophytic fungi might produce 77,450,000 unique secondary metabolites (110,800 × 699 per group) and estimating about half as many unique secondary metabolites per bacterial species, there would be perhaps 38,725,000 unique bacterial metabolites. However, considering studies that suggest ~ 90% of secondary metabolite biosynthetic capacity is silent or cryptic [33], the estimated endophyte-derived secondary metabolites on Earth might total 1.045 × 10^9^, or a billion potential endophyte secondary metabolites. ***Model-fitting****:* Estimates of endophyte species richness and NP potential could incorporate OTU data (e.g. see Fig. 1) into models of species discovery or species accumulation curves. These can be based on number of leaves sampled for endophytes [34] or published new species or OTUs [35]. Alternatively, endophyte OTU data can be estimated using frequency counts, rank species abundance distributions, or Poisson lognormal (log-log) fitting approaches and scaling laws [32, 36–40]. In the latter case, it has been argued that microbes in microbiomes closely fit the pattern where *S* (number of species) scales with *N* (number of individuals) where commonness (resampling) is constrained by scaling *N* ^*z*^ where *S ∼ N* ^*z*^ and 0.25 ≤ *z* ≤ 0.5 (and for microbes *z* = 0.38 while for macroscopic organisms *z* = 0.24), and globally *N*_*max*_ (number of individuals of the most abundant species) = 0.38 * *N* ^*0.93*^ r^2^ = 0.90 [32]. Empirically, results scale at *S* = 7.6 * *N* ^*0.35*^, r^2^ = 0.38. For endophytes, using estimated values of 10^4^ to 10^8^ endophytic bacterial cells per g of plant [41] plus ~ 10–100 fungal individuals per g, and an estimate of total Earth plant carbon (C) of 450 Gt [42] and assuming 0.43 g of C per 1 g plant matter [43], we estimate Earth’s bacterial endophyte individuals, *N* at 1.044 × 10^22^ to 10^26^, which with scaling laws results in an estimate of global endophytic bacteria species, *S* between 386 million and 9.7 billion and *S* for global endophytic fungal species between 34 and 77 million. These values produce not unreasonable estimates of numbers of microbial species per plant species, within the range of values summarized based on OTUs in Fig. 1 (i.e. for bacteria, 386 to 9700 million species divided by 300,000 plants = 1290 to 32,300 bacterial endophyte species per plant species – for example, similar OTU estimates in [44]; and for fungi 34 to 77 million species divided by 300,000 plant species = 113 to 257 fungal endophyte species per plant species). Extending the idea of endophyte secondary metabolite uniqueness per species-group as discussed above [11], there may be an estimated 124 million to 3.1 billion bacterial and 22 million to 50 million fungal secondary metabolites that could be expressed in cultures, and considering additional cryptic expression [33], up to 1.3 to 28.3 × 10^9^ potential endophyte secondary metabolites to be discovered.

**Table 2:** Estimating global plant microbiome holometabolomes using combinatorics

Table 1 considered endophytes independently, ignoring plant-microbe and microbe-microbe cooperation in NP synthesis. Here, we estimate secondary metabolism that may be more than the sum of its parts through *in planta* multi-species co-synthesis, syntropy, and synergistic biosynthetic pathways. We first estimate the global number of endophyte communities, then these communities’ additive and synergistic secondary holometabolomes ***Estimating the number of distinct endophyte communities on Earth***: While the theoretical number of possible combinations of endophyte species within a plant would be 2^*n*^ for *n* endophytes, so that a plant that can host 500 species of endophytes would have 2^*500*^ possible endophyte communities, real life does not include all possible combinations. Instead, if we calculate the possible unique endophyte sets of size 500 from amongst a set of *m* unique microbes, we can use the binomial coefficient and take *m* choose *k*, with *k* = 500, then solve for *m* based on the number of plant species or plant individuals on Earth. Given that *m* choose *k* = *m*!/*k*!(*m-k*)!, and assuming a limit of 300,000 combinations (one per plant species) we calculate *m* is between 502 and 503, meaning that the effective set of unique microbes per plant species would be just 2 or 3 under this endophyte set size of 500 per plant. Note: based on Table 1, we estimated from 113 to 257 fungi per plant species and from 1290 to 32,300 bacteria per plant species, which translates to effectively just over 3 unique fungi per plant species, and no unique bacteria per plant species. If plant individuals rather than plant species are better units for analysis, given the observed variance in endophytes across plant geographic ranges and predicted horizontal exchange of endophytes and we estimate an average of 50,000,000 plant individuals per species, there could be 15 trillion endophyte combinations on Earth, with as many as 6 to 7 unique endophytic fungi or over 3 unique endophytic bacteria per plant. ***Estimating endophytic holometabolomes on Earth***: Several studies have demonstrated the importance of plant-microbe and microbe-microbe shared metabolic pathways involving intermediate metabolite provision or positive regulatory cues [45]. Plant species diversity is positively associated with bacterial and fungal diversity and metabolism [25, 46], but how much of this is merely additive versus synergistic or cooperative? Results from OSMAC and co-culturing studies that show perhaps 90% of endophyte secondary metabolites depend on *in planta* conditions [28, 47, 48] pointing to *in planta* metabolic synergism. Combinatoric models have been helpful for exploring metabolism and biochemical space [49, 50], but these have seldom been applied to understanding synergies between microbes [51, 52]. Biosynthesis of secondary metabolites is particularly amenable to combinatoric synergy because it functions modularly through extending polymer backbones: -CH2-(C=O)- units for polyketides, C5 isoprene units for terpenoids, and non-ribosomomal peptides, that later generate diverse chemicals with the assistance of tailoring enzymes. Recent combinatoric experiments on simple microbiomes also indicate that higher-order interactions in which each species impacts interactions among other species, at least for 2-way and 3-way interactions are widespread in a 5-species microbiome [52]. Here, we will apply a similarly small interaction network for endophytes and consider the holometabolome of a small localized section of plant tissue containing 5 interacting organisms: the plant, two fungal species and two bacterial species, that can interact and cooperate molecularly at close range. In this example, 2^*n*^ interactions could occur for *n* species. If each pair of these 5 species participates in one biosynthetic synergy leading to an additional metabolite, there would be *n* choose *2* = *n*!/*2*!(*n-2*)! = 10 unique metabolites arising synergistically for this interaction. Within a plant species, if a portion of its endophytes (e.g. 100) participated in these synergies with each of the 10 novel products synthesized once, 100 / 5 = 20 such products would be generated, adding 6 million new secondary metabolites to the global tallies discussed in Table 1, or, at the upper limits, considering individual plants to have distinct communities and synergies, there could be 300 trillion unique *in planta* synergistic products on Earth. However, this value likely includes extensive redundancy, which is difficult to estimate without further empirical data, or models such as the deep learning models described in Table 3.	

This review will not present an exhaustive catalog of plant-associated microbes or NP chemical structures, which have been reviewed elsewhere [15, 73–77]. Nor will we cover detailed methodologies for extracting and analyzing endophyte secondary metabolites covered elsewhere [9, 78–80]. Instead, this review will present a novel analysis of the untapped potential of plant endophytic microbiomes for NP discovery, describing the breakdowns in signaling that lead to endophyte secondary metabolite silencing and upcoming breakthrough methods including deep learning. We describe recent progress in identifying hidden endophyte NPs through heterologous expression experiments [81], methods of unsilencing genes in endophytes [82] especially including co-culturing and condition-modification [28, 83]. We then highlight breakthrough approaches and strategies needing more attention, including systems biology methods [84, 85] integrated with big data mining and deep learning [56] from an *in planta* perspective. Specifically, we illuminate recent breakthroughs in artificial intelligence-based methodologies; particularly deep learning applied to multiple phases of the discovery pipeline and multi-omics *in planta*. We will finish by outlining a new, integrated pipeline – a systematic, interdisciplinary approach using computational learning – that promises to “wake the sleeping giant” of endophyte NPs.

### How much promise do endophytic microbiomes hold for natural product discovery?

Plant microbiomes may be one of the most promising and underdeveloped groups of organisms for natural product discovery, due to their long-evolved intimate interactions serving in chemical defense of plants [86–88]. For example, studies thus far on phyllosphere (i.e. above-ground microbiota) and root-associated microbiota have shown that endophytes provide bioactive secondary metabolites with unique structures such as Fusarihexin A & B, Pestalactams A & B, and polysaccharide DG2 [89–92]. But could they hold more promise for NPs than free-living microbes, as has been suggested [20]? This rhetorical question has practical importance: if endophytes do not hold exceptional promise as a source for novel NPs, it is pointless to invest exceptional effort to overcome the inherent challenges of their low culturability and high levels of silent gene clusters [93–95].

Answering this question requires consideration of how endophytic microbiota are distinct as a group. Once established in plant tissues, microbiome endophytes, in contrast to pathogens, can no longer increase their fitness by increasing biomass beyond the limited plant tissue growth, and instead can increase their fitness by switching their investment to benefits for the plant through increasing plant growth and synthesizing additional defense compounds [48, 84, 96, 97]. Plants and their microbiomes are distinctly limited in their options for escaping hostile interactions by means other than chemical innovation. Hence, endophytes show increased investments in defense roles, such as antiherbivory and antiviral activity, compared with free-living microbes [98, 99] ultimately showing enhanced directional or positive selection on defense compounds [87, 100], whereas within the confines of plant tissues their biomass investment is downregulated by the plant [101]. Furthermore, endophytes that proliferate mainly (or solely) within hosts will have enhanced drift or bottleneck and accelerated evolution [102–104] enhanced by phases of high local or vertical transmission [2, 15, 105, 106]. In addition, long-term interactions within plants likely places evolutionary pressure specifically at the level of molecule-to-molecule interactions and pathway-to-pathway interactions, enhanced by the large and complex plant genome [104]. For example, some endophytic fungi produce plant hormones (gibberellins and indolacetic acid) to promote host plant growth [97], and others synthesize plant-like defense compounds [101], famously including Taxol. For long-associated plant microbiome consortia, primary metabolism may decay, while secondary metabolism may be enhanced, sometimes on supernumerary chromosomes [107] or defense plasmids [108]. Thus, these distinct conditions in which endophytes have evolved should increase their secondary metabolite diversity. If so, why then do past surveys [109] suggest only ~ 5% of current medically relevant compounds are from endophytes? We explore answers to this question below, especially under-cataloging due to a focus on culture-based methods rather than analysis of the plant microbiome in situ or *in planta*.

### Hyperdiversity and its effects on holobiont metabolism *in planta*

Estimating the taxonomic and functional diversity of plant microbiomes is critical because species and strain diversity are believed to predict secondary metabolite diversity [110, 111]. To date, we lack a systematic census of global plant microbiome secondary metabolite diversity. A recent meta-analysis suggests complex evolutionary and ecological forces may influence the endophyte assemblages [112] and another recent study suggests adaptive matching drives diversification of plants and endophytes [104]. Therefore, in this section we illuminate key empirical studies showing the hyperdiversity of fungal, bacterial, and viral inhabitants of plants (Fig. 1) and present a new estimate of global endophyte diversity (also see Table 1).

#### Endophytic fungi are ubiquitous and hyperdiverse

Fungi appear to be the dominant microbial inhabitants, in terms of culturable biomass, in plants [113], and hence, likely the most prolific sources of endophyte NPs. Evidence of fungi in fossilized tissues of plants from ~ 460 million years ago may explain why fungi have diversified to all plants in all habitats studied to date [21]. Reports describing endophytic fungi in the tropics as “hyperdiverse” [25] have raised much interest in drug discovery. For example a seminal culture-based survey showed 418 endophyte morphospecies (~ 347 genetically distinct taxa) isolated from 83 healthy leaves of just two plants, *Heisteria concinna* and *Ouratea lucens*, in a tropical forest [25]. Despite these and other surveys [112], most of the world’s fungal endophyte taxonomic diversity – and therefore NP diversity – is uncharted. Clearly, fungal diversity estimates are wide-ranging and depend on census approach: culture-based studies suggest there may be ~ 5 to ~ 350 fungal endophyte species per plant, while culture-free amplicon-based deep sequencing based approaches, focused on 18S or ITS rRNA genes, suggest there may be ~ 40 to 1200 fungal endophyte species per plant (see references in Fig. 1).

Species counts alone do not estimate functional or metabolic diversity; specific fungal endophyte clades differ in roles, and therefore biosynthetic capacity. For example, fungal associations can be foliar, systemic, or root-limited and will differ in roles accordingly. Taxonomically, most endophytes fall into the non-balansiaceous group (non-grass endophytes), which include diverse hyphae-forming Ascomycota (the dominant phylum of fungal endophytes), Basidiomycota, and Glomeromycota [114]. Many of the common genera, such as *Acremonium, Alternaria, Cladosporium, Coniothyrium, Epicoccum, Fusarium, Geniculosporium, Phoma*, and *Pleospora* are ubiquitous [115] with some groups dominating in the tropics (Xylariaceace, *Colletotrichum*, *Phyllosticta*, and *Pestalotiopsis*) and others common to both tropical and temperate climates (e.g. *Fusarium, Phomopsis*, and *Phoma*) [115, 116]. Biosynthetic capacity relevant to natural product discovery appears to be distributed broadly across these fungi. For example, a study of endophytic fungi with antitumor activity showed dominance of Ascomicotina (96%), but broad taxonomic distribution within this group, and others such as Basidiomycota (3%) and Glomeromycota (1%) [117]. The genera identified as antitumor compound-producing are broad (e.g. including *Pestalotiopsis, Aspergillus, Chaetomium, Fusarium, Penicillium, Alternaria, Phomopsis, Acremonium, Ceriporia, Colletotrichum, Cytospora, Emericella, Eurotium, Eutypella, Guignardia, Hypocrea, Periconia, Stemphylium, Talaromyces, Thielavia* and *Xylaria*) [117]. In contrast, Balansiaceous endophytes (or grass endophytes) are narrower taxonomically and include clavicipitaceous genera *Epichloë* and *Balansia*, with their anamorphs *Neotyphodium* and *Ephelis* predominating. Balansiaceous endophytes are notable for their vertical transmission with seeds and production of anti-insect alkaloids peramine and lolines, and the anti-vertebrate alkaloids lolitrem B and ergovaline [118]. In preparing this review, we found no comparative analysis of the classes of secondary metabolites or natural products grouped with endophyte tissue- or taxon-class, but presumably such patterns do exist.

There has not been a comprehensive model to estimate the diversity or richness of endophytic fungi, but an often cited calculation suggested there are 2–4 unique endophytic fungi per plant, which would suggest there are ~ 1 million species of endophytic fungi on Earth, based on an estimated 270,000 plant species [11]. However, these estimates predate next generation sequencing studies [119–122], and likely suffer from bias against non-culturable taxa. Thus, we have attempted to synthesize some of the recent sequence-based data on endophytic fungal diversity within plants at a taxonomic level most relevant for NP discovery (i.e. strain-level), integrating established models (e.g. Poisson lognormal) in Table 1. These provisional calculations suggest far more diversity than previous estimates, with possibly 34 to 77 million endophytic fungal species and 10 to 20-fold more strains on Earth with capacity to synthesize 22 to 50 million biosynthetic gene clusters (BGCs) based on pangenome-level BGC analysis.

#### Endophytic bacteria are also ubiquitous and hyperdiverse

Bacteria are the other dominant and diverse microbes associated with plants, providing additional metabolic and biosynthetic capacity. Recent reviews have presented endophytic actinobacterial secondary metabolites in depth and described key interactions and metabolites in this group [6, 123]. Taxonomic profiling studies have tended to focus on crops, fruits and vegetables [124–126], or forest tree foliar endophytes [127] and cold adapted plants [122]. Nevertheless, endophytic bacteria are poorly known, despite the fact that bacteria are the most speciose and metabolically diverse domain of life, with perhaps 1 trillion species [32]. Bacterial endophyte diversity may be far more under-cataloged than endophytic fungal diversity due to the small size, low biomass, less clear ecological roles. However, some studies suggest bacteria are ubiquitous, colonizing all parts of plants as inter- and intra-cellular endophytic bacteria living in roots, stems, shoots/leaves, and vascular tissues [41, 128–131], as well as foliar epiphytes on leaf surfaces [132–134], rhizosphere associates on root surfaces and the more well-studied nodule-forming root endophytes (e.g. rhizobia in legumes) [135–137]. While endophytic bacterial diversity can be extremely high (e.g. 31,952 OTUs at 97% similarity) [44], typically, the number of distinct bacteria per plant ranges from 10 to 200 for culture-based studies and from 20 to 600 from amplicon sequencing-based studies (see references in Fig. 1). While no current models exist to estimate bacterial endophyte diversity, based on extant 16S rRNA surveys of bacterial endophytes and the framework used above for fungi, we estimate there may be perhaps 386 to 9700 million bacterial endophyte species on Earth, with perhaps 124 to 3.1 billion biosynthetic gene clusters (Table 1).

#### Endohyphal bacteria may enrich endophytic fungal diversity and metabolite synthesis

Endohyphal bacteria (EHB) live within free-living and endophytic fungi, adding to their biosynthetic capacity, function and regulatory complexity [62, 63, 67, 138]. Far from being rare, EHB appear to be widespread [64], potentially protecting the plant and endophytic fungi from pathogens [65] and interacting with plant hormones [66]. EHB have been described as the prokaryotic modulators of host fungal biology in hyphae of endophytes in many plant tissues and across many plant lineages [139, 140]. This endosymbiotic association was first detected inside the mycelium of mycorrhizal fungi wherein mycorrhiza helper bacteria were associated with the fungal nutrition transport [62]. A remarkable example is the ectomycorrhizal fungus, *Amanita muscaria*, and a mycorrhiza helper bacterium, *Streptomyces* strain AcH 505. Strain AcH 505 produces both fungal growth-stimulating compounds (e.g. auxofuran) and compounds that suppress plant-pathogenic fungi, and alters gene expression in *A. muscaria* [63]. In some cases, EHBs may enhance stress tolerance of plant and fungus, production of phytotoxins and regulation of host reproductive machinery [61], influence the ecology of plant endophytes [64], or confer other types of protection to the host fungus or plant [65]. Although these bacteria play important roles in modulating the secondary metabolism of their host fungi, this is still poorly understood.

#### Viruses of plants and endophytes impact the holobiont metabolism

Viruses are widespread and diverse pathogens of plants, fungi, and bacteria and can impact their host populations and alter host SM biosynthesis [141–144]. Hypovirulent viruses and phage are of special interest for potentially serving to regularly unsilence NP clusters [145–147]. We consider three important types of viruses: (1) mycoviruses, i.e. viruses that infect fungi and show low virulence; (2) bacteriophage of endophytic bacteria and endohyphal bacteria; and (3) latent plant viruses. Mycoviruses are diverse and classified into seven families of double-strand RNA (dsRNA), single-strand RNA (ssRNA) and single-strand DNA (ssDNA) [70, 141, 148]. These hypovirulent mycoviruses have been diagnosed from all classes of endophytic fungi [142]. However, mycovirus diversity and host-specificity is still poorly understood, and the role of mycoviruses is poorly understood. For example, mycoviruses in the endophytes of *Ambrosia psilostachya* and its parasite *Cuscuta cuspidata* were shared between different fungi [149] suggesting they might not be specific to a single fungal taxon. In contrast, endohyphal viruses of related endophytes of Pine, *Diplodia scrobiculata* and *D. pinea* and appear not to be related [150]. Nevertheless, mycovirus species richness appears to be vast, with viruses identified in over 30–80% of fungal species [70]. Specialized mycoviruses that may impact fungus-plant interactions. A notable example is the fungal endophyte *Curvularia protuberate* of the tropical panic grass *Dichanthelium lanuginosum* in which its mycovirus allows the plant to grow at high soil temperature [68].

Bacterial viruses, or bacteriophage (phage), are hyperdiverse with perhaps 10 or more estimated unique phage per species of bacteria [151–153].. However, little is known about of phage that specialize on endophytic bacteria. Nevertheless, they almost certainly affect endophytic and endohyphal bacterial fitness, population dynamics, and aspects of secondary metabolite production that involve these bacteria.

Plant viruses, especially latent or persistent plant viruses that remain asymptomatic for extended periods of time, including Endornavididae, Partitiviridae, and Luteoviridae, are diverse and ubiquitous [154–157]. Numerous studies suggest that together, plant viruses may impact plant resistance to infectious and beneficial bacteria and fungi, and may impact plant interactions with and colonization by endophytes [154–157]. Detailed studies of the impacts of plant viruses on plant secondary metabolism [158, 159] suggest ways in which the plant holobiont (including its resident endophytes) may shift gene expression, proteome, and metabolome, resulting in altered holobiont NP profile [155].

### Are plant microbiome communities greater than the sum of their parts?

**Fig. 2:**
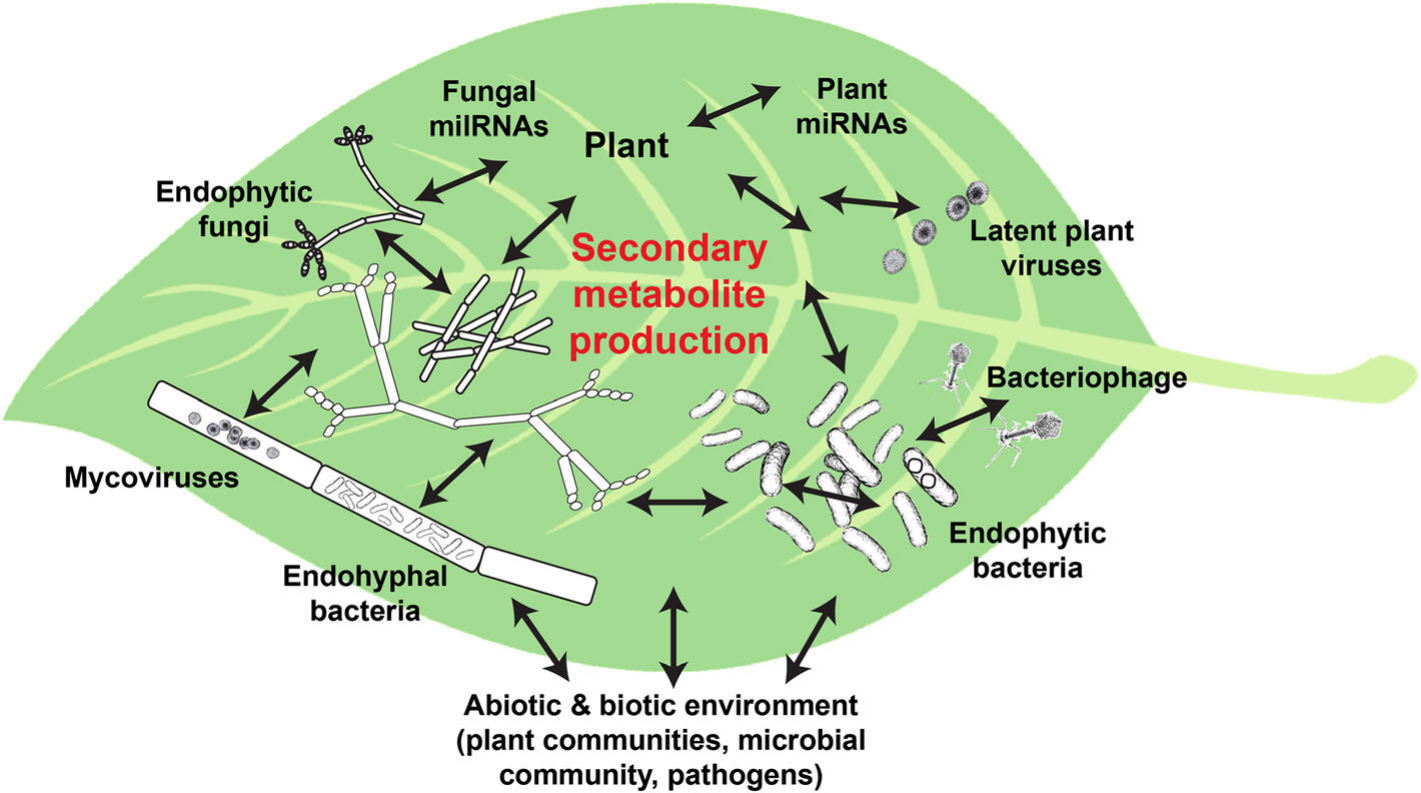
Schematic of the plant microbiome showing *in planta* interactions leading to multipartite biosynthesis and regulation of endophyte-plant (holobiont) secondary metabolites

Much of secondary metabolism in cells contributes to the “holometabolome” (i.e. the net metabolome of the holobiont) additively. However, many studies suggest that *in planta* endophyte community interactions and regulatory cross-talk (see recent review [140]) that may influence secondary metabolite synthesis [45, 160–162]. Some of these major interactions within plants, such as plant-endophyte, fungi-fungi, fungi-bacteria, fungi-EHB, fungi-mycovirus, bacteria-phage, and miRNA and small-molecule signals, are shown in Fig. 2. Several studies suggest a portion of the holometabolome may arise through provisioning of substrates, such that secondary metabolism is not merely additive, but instead is greater than the sum of its parts. For example, endophytes may metabolize secondary compounds from the host, or the host and endophyte may share parts of a specific pathway – although this is not well-known [161]. One example of this is the putative combined synthesis of cardiotoxin by endophytic *Burkholderia* spp. and plants [123, 163, 164]. Generally, most evidence for cooperative exchange comes from laboratory co-cultivation studies, suggesting fungi-fungi and bacteria-fungi interactions may impact SM production [165, 166]. Indeed, it is the rule, rather than the exception in microbial communities that multiple species may exchange a plethora metabolites – hence, classical models of inter-species metabolite exchange [167]. There has been speculation about the role of horizontal gene transfer as a key factor in the apparent convergence of endophyte and plant metabolites [168], but to date, this question has not been thoroughly examined. Co-regulation of independently evolved BGC homologs in plants and their microbes has also been described [169], but remains poorly understood. Secondarily, endophytes may prime the host plant’s defense via ethylene-jasmonic acid transduction, mediators of biotic and abiotic stresses and ROS, modulating plant receptors for chitin and flagellin [61, 140], although this is better known for plant-pathogens than endophytes and similar studies for mutualistic endophytes are lacking.

Empirical and theoretical analysis of endophyte taxonomic and functional diversity should inform bioprospecting strategies and be particularly helpful for identifying novel *in planta* communities that might produce novel natural products. However, few studies have examined this. One study estimated at least one unique endophyte community per plant species [2]. We re-estimate this in Table 2 using a combinatoric approach and suggest there may a range of 1 community per plant species to 1 community per plant individual or 300,000 to 15 trillion combinations on Earth. To evaluate global *holometabolome diversity*, we considered both the sum of endophyte metabololic potential alone and estimated possibly 1.3 to 28.3 × 10^9^ metabolites (Table 1) and then we additional synergistic metabolism by considering only subcommunities within plants, and estimated these could add between 6 million to 300 trillion unique *in planta* synergistic products on Earth (see Table 2). Co-regulation and downregulation will arguably reduce the biosynthesis overserved at any time, so these estimates would reflect long-term capacity under a variety of environmental conditions and triggers.

### Chemical diversity in the plant microbiome: a universe of natural products

Compounds from endophytic consortia likely traverse the sphere of possible natural products. Chemical diversity, or chemical space (all molecules that might exist) has been estimated theoretically at > 10^60^ small compounds < 500 Da. Natural products occupy a part of this theoretical space, mostly falling into four categories of secondary metabolites (alkaloids, terpenoids, phenylpropanoids, and polyketides). Current curated natural compound databases such as the Dictionary of Natural Products and Super Natural II [170], which include over > 325,000 natural compounds with only perhaps about 5 to 10% of known bioactive products come from microbes [13, 171] with perhaps half from Actinomycete bacteria (particularly *Streptomyces*), and a growing proportion from fungi, but only a few chemical compounds recognized from endophytes. From 2014 to 2017, a total of 224 novel compounds were recognized from endophytic fungi [73]. Estimates of all possible undiscovered natural compounds on Earth could range from near the current asymptote of discovery (i.e. with only 25,000 more to be discovered) [172] up to one per undiscovered microbe [173], which, with 99.999% of Earth’s microbes undiscovered [32], might yield 5000 to 2 million novel NP-derived drug candidates. But drug chemical space is much smaller than natural product space due to the limitations of oral administration and pharmacokinetics – following Lipinski’s rule of five. Conversely, despite known natural products being a tiny portion of all theoretical compounds, they contribute more than half of FDA approved drugs likely because evolutionary forces promote natural compounds with specific bioactivities.

However, the curve of natural product discovery appears to be leveling off [172]. Arguably, one reason for the leveling is that we have reached the limits in methodology and screening approaches that focus mostly on the small proportion of microbes that can be easily cultured under laboratory conditions. For example, analyses of secondary metabolite libraries suggest that while we have reached some limits in examining planar compounds (2-dimensional or sp^2^-hybridized double bond-rich) that are effective in interacting with similar targets (e.g. kinases), we have under-examined the richer drug potential of diverse 3-dimensional compounds (e.g. those with fewer aromatic rings and more sp^3^-hybridized single bond carbons with higher stereochemical center diversity) that will in theory have vastly greater target richness (e.g. protein-protein or transcription factor) [173]. Some of these may be expressed only under special conditions. Indeed, genome analysis has uncovered universal microbial processes to down-regulate or silence biosynthetic gene clusters [174]. In fact, genome mining studies suggest 92–96% of fungal secondary metabolite biosynthesis is routinely turned off [175, 176] through epigenetic regulators and absence of triggers from other organisms [177], presumably to reduce energetic costs during times when the products do not add to fitness. Furthermore, as argued in Table 2, chemical complexity may depend on community interactions that transform compounds [3], sometimes through enzymes or shunt metabolites (e.g. acetyl-CoA, shikimic acid, mevalonic acid, 1-deoxyxylulose-5-phosphate, in alkylation, decarboxylation, aldol, or Schiff base formation) [178], via natural biotransformation or bioconversion. Even Taxol biosynthesis seems to depend on microbe-microbe, microbe-plant, and abiotic factors [179, 180]. Cooperative biosynthesis has been described extensively in microbe and microbe-host systems [71, 181, 182]. Several studies suggest endophytes can in some cases can directly synthesize plant-like metabolites [183].

Studies of bioactive compounds from fungal endophytes of leaves and roots [184–187] show that while only a few strains have been extensively studied, typically each has several novel compounds (e.g. Li et al. 2018 reviewed 224 compounds from 109 endophyte strains). The taxonomic distribution of fungal endophyte derived chemical compound synthesis is dominated by Ascomycota (~ 97%) (with classes Sordariomycetes ~ 40%, Dothideomycetes ~ 31%, Eurotiomycetes ~ 24%, include notable pathogens as well as endophytes), with some Pezizomycetes and Agaricomycetes, and also Basidiomycota (~ 2%), and Mucoromycota (~ 1%) with the most richly represented compound-producing strains belonging to *Aspergillus*, *Penicillium*, *Pestalotiopsis*, followed by *Fusarium*, *Phomopsis*, and *Alternaria* [73, 117]. Notably, 5 of 14 strains of *Pestalotiopsis* produce the cancer drug Taxol. Similarly, recent studies of anti-cancer compounds isolated from endophytic fungi showed novel alkaloids and nitrogen-containing heterocycles (> 27 new compounds including penicisulfuranols, penochalasins, aspergillines, etc.), polyketides (> 25 new compounds including phomones, rhytidchromones, allahabadolactones, etc.), terpenoids and steroids (> 18 new compounds including rhizovarins integracides, etc.), quinones, phenylpropanoids, and esters (> 20 new compounds including versicoumarins, versicolols, pestalotrioprolides, etc.), and other classes of compound (> 35 new compounds including muroxanthenones, etc.) [73]. Another review showed compounds from endophytic fungi of similar taxonomic breadth having potentially activity against neglected tropical diseases (including compounds Citrinin, palmarumycins, Cochlioquinone, Grandisin, Altenusin, Pullularins, Pestalactams, Viridiol, Phomoarcherins, etc.) [188]. Further reviews have highlighted the wide array of therapeutics isolated from endophytes that mimic therapeutic plant-derived secondary metabolites, e.g. antioxidants (Lapachol, Cajanin stillbene acid, Resveratrol, Rutin, Phillyrin), antihypercholesteromics (Rosuvastatin, Piperin, Chartarlactams, Phenlspirodrimanes, Lovastatin), antidiabetics (2,6-di-tert-butyl-p-cresol, Berberine, Cajanol, Aspergillusol A, Rohitukine, Helvolic acid), and further compounds identical to plant-derived anticancer compounds (Taxol, Hypericin, Vincristine, Vinblastine, Camptothecin, Podophyllotoxin, Kaempferol, Azadirachtin, Rohitukine) [189–191] possibly as an ecological survival strategy [168]. In a few cases, research shows endophytic compounds to be exceedingly rare, yet especially useful medically, such as the unique mellein compounds of *Aspergillus flocculus* (Tawfike et al., 2019). From 2010 to 2017, 65 metabolites from endophytic fungi were identified as antimicrobial and anticancer agents with unique compounds such as Solamargine (alkaloid), Piperine (alkaloid), Cajanol (flavonoide), Vinblastin and vincristine (alkaloids), Forskolin (alkaloid), Homoharringtonine (alkaloid), Chrysin (flavonoid), and have antimicrobial and anticancer activities [84, 191–193].

Amongst bacterial endophytes, Actinomycete bacteria have been studied extensively, especially *Streptomyces*, *Micromonospora*, *Polymophospora*, *Jishengella*, and *Actinoallomurus* which produce many remarkable bioactive compounds including highly modified alkaloids (diketopiperazines, lansai, spoxazomicins, dihydrooxazole alkaloids, spoxazomicins, pyrazine), peptides (such as cyclotetrapeptides), a wide array of polyketides (such as glycosylated and prenylated antibiotic coumarins, butyrolactone antibiotics, cedarmycins, pteridic acids, clethramycin, efomycin M, salaceyins, lorneic acid, stipitatic acid, secocycloheximides, maklamicin, linfuranones, germicidin, actinoallolides, alnumycin, lupinacidins), terpenoids (such as kandenols), and mixed synthesis metabolites (such as indolosesquiterpenes, xiamycin B, indosespene, sespenine, celastramycin, and trehangelins) [171].

Together, these studies show an increasing universe of natural products with novel bioactivities compounds from fungal and bacterial endophytes, even in the absence of *in planta* inputs such as precursors and regulatory molecules, or environmental cues. It remains unclear if this universe will continue to expand, or if the predictions in Table 2 will ever be realized, but we argue the primary challenge will be harnessing new potential from the vast unculturable majority of microbes.

### Isolation is the problem

Isolating and culturing plant microbiome species to uncover their biosynthetic capacity is a poor strategy for two reasons; first, most endophytes cannot be grown in culture, and second, most endophytes will not express many secondary metabolites outside the host plant tissue or environmental niche. The apparent failure of culturing for most microbiota within plants makes sense given the long association of these organisms and the widespread tendency of symbionts to lose the capacity for traits needed to live outside the host, due to relaxed purifying selection on those traits. Studies on the fungal endophytes that can be easily cultivated suggest taxa and their secondary compounds are tissue- and organ-specific, and seasonally, and geographically variable [15]. This pattern is likely mirrored by the even more host-adapted non-cultivatable endophytic fungi and bacteria, and likely translates to further hidden biosynthetic diversity. For example, one study showed high NP diversity from non-cultured 3409 endophytic bacteria, but only 1.6% of the identified BGC clusters matched any known BGC [194]. The new era of advanced sequencing and computation discussed in this review should result in a sharp rise in discoveries for these difficult-to-culture microbes. However, traditionally, culturing has been required to confirm and analyze natural compounds. This problem is one of the major breakdowns in the NP discovery pipeline: breakdown of microbe-host molecular exchanges makes plant microbiomes difficult to study.

Endophyte NP diversity is under-cataloged, even for culturable species, presumably because culturing methods fail to adequately supply *in planta* molecular signals required to unsilence BGCs [14, 195–201]. This observation derives from sequencing studies and metabologenomic analyses showing evidence of BGCs for products that are not detected in cultures. As a primary example, polyketide synthases (PKSs) and nonribosomal peptide synthetases (NRPSs), which are multifunctional enzyme systems that assemble many of the secondary metabolites from simple building blocks including carboxylic acids and amino acids [202, 203], show limited expression under laboratory conditions [204]. Extensive efforts have been made to unsilence such clusters [205, 206]. Most genetic manipulation methods attempting to control PKSs and NRPSs as multifunctional enzymes to regulate expression of BGCs rely on multi-target approaches not specific to a single secondary metabolite and display complex interactions.

In fungi, control is often regulated by chromatin-based mechanisms and histone acetyltransferases, deacetylases, methyltransferases, and proteins involved in heterochromatin formation [207, 208], thus, modifying the chromatin landscape through chemical modifiers can regulate secondary metabolite synthesis [111]. Specifically, many putative silent BGCs are located in the distal regions of the chromosomes in the heterochromatin which is controlled by epigenetic regulation [209]. However, these modifications can lead to unpredictable changes in expression of other genes [111]. This is true for the fungal blight pathogen, *Fusarium graminearum*, where increasing the expression of the heterochromatin protein homolog (HEP1) which plays an important role in the production of secondary metabolites. HEP1 influences expression of genes of aurofusarin with antibacterial/toxicological effects [210]. Other attempts at changing chromatin do not always unsilence cryptic fungal BGCs, since most secondary metabolite gene clusters remain silent by these approaches [211]. Many methods that include pleiotropic and pathway-specific approaches have had similarly limited effectiveness. For example, small-molecule elicitors released from plant hosts may affect endophyte SM transcription, many studies of endophytes grown outside plant tissues have used epigenetic modulators to attempt to activate the silent BGCs [212], with inconsistent results. Small molecule epigenetic regulators and in different expression-type strains of different PKS reduction states stimulated a variety of alternative VOCs [213], while heterologous expression experiments [81] and other unsilencing approaches [82, 214] have had mixed success.

*In planta* studies of the plant microbiome in situ, in contrast to studies of cultured endophytes, have revealed that broad gene expression derives from integrated, dynamic components of the plant-endophyte holobiont [215]. This integration of gene expression regulation may be ~ 460 million years old [21, 22], enough time for the evolution of cooperative synthesis of compounds and precursor supply (or regulation of degradation of precursors for secondary metabolism) [72], with the help of neighbors, such as the plant, other endophytic fungi and bacteria [61, 142]. Thus, breakdowns between endophyte and host metabolism, precursor supply, and signaling may drive biosynthetic gene clusters to be silenced as they are studied in culture. For example, studies show that endohyphal bacteria such as members of the Enterobacteriaceae, which may impact fungal gene expression [61–67], may diminish or change during culturing [216]. Clearly, expression of BGCs can be context-dependent Even simple variations in the growth medium such as pH, temperature, aeration, and light can change the level of transcription of BGCs [217]. This point is evident from co-cultivation experiments that provide interspecies signals for SM synthesis [218], and in vitro multi-endophyte array experiments [191]. In many studies, co-cultivation of endophytic fungi with their plant hosts led to the activation of formerly silent gene clusters [219]. Another missing signal in cultured endophytes may be small RNAs. These have been observed to transmit bidirectionally [220] as a mode of trans-kingdom cross-talk [221, 222] and may transcriptionally activate silent clusters or regulate translation in response to infection [223]. Indeed, fungi encode microRNA-like small RNAs (milRNAs) that may interact with other regulatory elements and affect transcription and post-transcriptional changes [224, 225]. Furthermore, miRNAs triggered by pathogens could unsilence endophyte fungi or unsilence plant signals directed at endophytes, that turn on genes for SMs. Some remarkable small RNAs in bacteria may impact hosts, and miRNAs from hosts may pass into endophytic bacterial cells and regulate their expression [223].

But why should endophyte BGCs be silenced during growth in culture? And why should plants down-regulate endophyte SM production except under specific conditions? The proximal cause of silencing in culture may be simple lack of signals or precursors, however, the ultimate evolutionary cause may be the need to redirect energy to growth [204]. Long-evolved intimate partners often chemically stabilize and control their interactions with neighboring organisms to coordinate or regulate growth [200] conserve energy and maintain the novel benefits of symbiosis.

### Past and current solutions to discover NPs from plant microbiomes

#### Approaches focused on cultivatable endophytes

Standard pipelines for endophyte NP discovery are powerful, but usually low-throughput [29]. Historically, prior to next generation sequencing, methods for discovering endophyte-derived natural products would involve (1) field surveys to extract plant tissues, (2) endophyte (bacterial or fungal) culturing (e.g. for fungal endophyte culturing, see [188]), (3) extraction and separation of compounds for analysis, (4) chemical analysis and dereplication using any of many classical techniques such as UV spectroscopy, infrared spectroscopy, mass spectroscopy (MS), and nuclear magnetic resonance spectroscopy (NMR) or more modern “on-line” hyphenated (i.e. coupled) approaches such as HPLC-NMR-MS (see [178], (5) and finally bioactivity assays and testing on cells/animals. To speed up drug discovery, the search for natural product extracts was largely supplemented from the 1990s onward with synthetic combinatorial chemistry approaches which create large compound libraries that can be tested using automated high throughput screening (HTS). However, this approach has proven to have limitations [178].

Simultaneously, some of the limitations of natural product discovery have been overcome by increasingly sophisticated standard methods. Key methods in use are pleiotropic approaches such as “One Strain – Many Compounds” (OSMAC), chromatin remodeling, ribosome engineering, or targeting global regulatory genes or phosphopantetheinyl transferases, approaches that are specific to BGCs such as heterologous expression, promoter exchange, refactoring, and cluster-situated regulators, and genome-wide targeting by reporter-guided mutant selection and elicitors [226]. The OSMAC approach, which centers on testing each isolated strain grown under a systematic array of culture conditions to increase the diversity of secondary metabolites produced has been one of the most effective NP discovery methods for culturable endophytes [28, 83]. In OSMAC, common modifications include high phosphate, modified media richness, pH value, temperature, salinity, metal ions, oxygen/aeration, or with addition of enzyme inhibitors [83, 227], or using UV mutagenesis, or with addition of plant or microbial extracts or cells or under co-cultivation, or affixed to various surfaces (i.e. as biofilms), or epigenetic modifiers (e.g. DNA methyltransferase inhibitor, histone deacetylase inhibitor, biosynthetic precursors). OSMAC’s promise as a method ultimately derives from simulating not only abiotic but biotic plant niche-like triggers for endophyte gene expression.

Cocultivation approaches likely function in the same way, providing biological signals to modify gene expression [218]. In a remarkable recent example of co-culturing, Taxol gene expression was restored in *Aspergillus terreus* by culturing it in the presence of *Podocarpus gracilior* (African fern pine) leaves [228]. Similar triggers occur in heterologous expression experiments, for example, in Aspergilli [229]. Fungal-*E. coli* shuttle vectors (FACs) have been used to identify SMs and gene clusters combined with LC-MS (i.e. FAC-MS) that may force expression of silent clusters [230]. Using regulators and promotors can help researchers to control the level of gene expression. For example, in the rice fungus *Monascus pilosus* the monacolin K and terrequinone A gene clusters from *Aspergillus nidulans* were successfully overexpressed in *Aspergillus oryzae* using a constitutive active pgk promoter [231]. Genetic methods that have been used to unsilence BGCs include heterologous host ribosome engineering [229, 232], insertion of constitutive or inducible promoters [233], reporter-guided mutant selection [234], and interfering in the condensation state of the genomic DNA by inactivation of DNA-modifying enzymes [213]. Manipulation of genes involved in microorganism development is another promising unsilencing method [235]. Finally, for bacteria ﻿there are high-throughput methods not involving genetics, like high-throughput elicitor screening with imaging mass spectrometry (HiTES-IMS) that promise to induce the silent secondary metabolome in response to ~ 500 conditions [47]. Yet, most of these methods are either low throughput, or work only for culturable microbes.

#### Approaches using next generation sequencing, comparative genomics, genome-scale metabolic models, and metabolic network modeling

High-throughput sequencing and bioinformatics combined with other newer technologies over the past 15 years have been instrumental in identifying unculturable endophytes communities and opening new horizons for expression of silent BGCs. For example, through comparative genomics, we now know that much of the chemical diversity in microbes derives from enzyme clusters, or biosynthetic gene clusters (BGCs) that are conserved across many species, such as the tailoring enzymes consisting of non-ribosomal peptide synthetases (NRPS), polyketide synthases (PKS), and terpene synthases (TPS) and terpene cyclases (TCs), phenytransferases (PTs) along with associated genes for regulation, uptake of substrates, and transport and secretion of products [236, 237]. Some are also synthesized, carried, or tailored by post-translationally modified peptides (RiPPs). There are other specialized or taxon-specific BGCs, but because these often remain silent or expressed at very low levels under laboratory conditions, it is often difficult to confirm that the genes are functional. Thus, many strategies to discover NPs from microbes begin with bioinformatic prediction of BGCs from genomic data, followed by experimental induction of predicted silent biosynthetic pathways through genetic engineering or an array of methods discussed above.

Continuing efforts at database and software development have been especially important in refining the search for plant microbiome-derived NPs. Various ‘older’ software include untargeted genome mining approaches using the ClustScan software and ClustScan Database (CSDB) [238], ‘Database Of BIoSynthesis clusters CUrated and InTegrated’ (doBISCUIT) [239] which identifies clusters involved in tailoring enzymes, and ClusterMine 360, which includes 200 PKS & NRPS [240]. Other older approaches include the software ‘Secondary Metabolite Unknown Region Finder’ (SMURF) [241] which is a web-based HMM tool to identify conserved domains in PKS, NRPS, hybrid-PKS/NRPS and terpenoid gene clusters in fungi and the updated Joint Genone Institute (JGI) ‘Integrated Microbial Genomes - Atlas of Biosynthetic gene Clusters’ (IMG-ABC) for identification of gene clusters [58]. An increasingly useful database is ‘The Minimum Information on Biosynthetic Geneclusters’ (MIBiG) [242, 243]. These approaches have been used for phylogeny-based BGC discovery [244], which has been shown to be effective in identifying inhibitors of multidrug resistant pathogens [245].

However, many of these tools have been superseded by or integrated with leading current comprehensive toolset and databases for genome-wide annotation and analysis of BGC, the ‘antibiotics & Secondary Metabolite Analysis Shell (antiSMASH), with current version 5.0 [55, 110]. antiSMASH works as a web-server or downloadable software, and primarily runs NCBI BLAST+, HMMer 3, Muscle 3, FastTree, PySVG and JQuery SVG, along with many other previously published secondary metabolite analysis tools. Genome-wide metabolic models (GEMs) can enhance these approaches, for example with the ‘Reconstruction, Analysis and Visualization of Metabolic Networks’ RAVEN 2.0 software [246, 247] and MetaFlux [248] which has been integrated into the comprehensive toolset Pathway Tools [54]. Of particular interest for community metagenomic holometabolism data from *in planta* studies and Pathway Tools v2.30’s multi-pathway diagrams (pathway collages) and its new algorithm for generating mechanistic explanations of multi-omics data [54].

Network-algorithm-based software can improve the predictive power of these genome mining approaches by incorporating ecological interactions [216]. For example, secondary metabolite gene cluster similarity networks [249], and network simulation models have been useful in studying metabolic production during interaction [250]. These approaches can be combined with metabolic modeling approaches, such as flux-balance models [167] with predictive mechanistic frameworks that predict core metabolism. Metabolic interactions in microbial co-cultures are perhaps best modeled this way, with the Metabolic Support Index (MSI) used to predict the microbial interactions in a co-culture and understand which microbe receives maximum benefit from the interactions [251]. The MetQuest software explores possible benefits derived by microorganisms from interactions in a community [252], although such results require follow up using physiological experiments. Biokinetic models have also been developed for interspecific interactions among microorganisms sharing substrates in an ecosystem [253]. Single-cell analysis could augment our understanding of endophyte metabolism [192], particularly with the addition of context-specific transcriptomics. Remarkable insights have been made from transcriptomic studies. For example, fungal regulation appears to be conserved during SM production [72] and can be confirmed via *in planta* transcriptomics [254]. Further promising transcriptomic methods that can be integrated with *in planta* strategies include Iso-seq (long read transcript sequencing), illuminating alternative splicing in Taxol production [255], and miRNA target transcriptome-mining [256].

### More powerful solutions

#### Deep learning for global plant microbiome NP bioprospecting

Despite our general predictions of potential plant endophyte diversity (Table 1) and endophyte community (i.e. microbiome) diversity (Table 2), the true distribution of endophytes and their potential natural products remains largely unknown [112]. To focus future endophyte bioprospecting requires a new, rigorous framework to guide strategic field sampling. NP exploration strategies must also be sensitive to threatened species and habitats. Machine learning and deep learning approaches, which are defined and described in Table 3, offer an exciting option.

**Table 3:** Machine learning and deep learning approaches for plant microbiome-based natural product discovery

For predicting features of data that are too large to be completely sampled, one of the most promising approaches is computational learning or artificial intelligence, including machine learning and deep learning. These approaches deal with the problem of having an incomplete model to characterize unseen data, by evaluating diverse competing models on a set of training data. In other words, these approaches complete tasks without explicit instructions using patterns (models) learned from the training data. Specific machine learning approaches include Random forests, Hidden Markov Models, hierarchical cluster analyses, and support vector machines. Deep learning is a type of machine learning that handles additional complexity by using layers of data transformations. Specific deep learning approaches use convolutional neural networks where each layer learns from other, previous layers which are called hidden layers. One common framework for building such tools is the well-supported R Interface ‘H2O’ Scalable Machine Learning Platform (GitHub at h2oai/h2o-3) [53]. For ***global endophyte NP bioprospecting***, we can integrate phylogenomic deep learning and genome-wide metabolic model deep learning frameworks. For example, using Pathway Tools v.23.0 [54] integrated with MetaFlux in antiSMASH [55] and DeepBGC [56]. For ***predicting the chemical structural diversity of endophytes***, we can interface the approaches above into chemoinformatic and drug discovery deep learning frameworks. For ***discovery of in planta unsilencing triggers – waking the sleeping giant,*** we can integrate experimental system data, OSMAC, and multi-omics data (e.g. from data mining amplicon sequencing, shotgun sequencing, metatranscriptomic sequencing, and metabolomics) Table 1**Inset:** Recent trends in peer-reviewed studies with keywords/title “endophyte”, “endophyte and natural product”, showing limited increase, whereas studies on “deep learning”, “multi-omics” are steeply increasing	

Ideally, machine learning or deep learning frameworks could begin to predict plant microbiome distribution patterns in the context of environmental niches, while also predicting endophyte-derived natural products, thus, replacing comprehensive, global-scale, molecular surveys of plant microbiomes, which are challenging for all but a few clades.

Initial training data sets could capitalize on existing the growing array of genomic, phylogenomic, and multi-omic surveys, particularly those with metabolomics from natural plant tissues, i.e. the holotranscriptome and holometabolome. To increase training data, complementary, strategic multi-omics studies could be performed based on identified hotspots. These data can be combined with network co-occurrence analysis, metabolic cooperation or complementarity analysis, and community biosynthetic pathway analysis [216, 249, 250, 252, 257].

Several machine learning and deep learning software approaches are already in use for natural product discovery. For example, ClusterFinder [258] uses machine learning for known (curated) and unknown classes of BGCs, trained using a hidden Markov model-based probabilistic algorithm. DeepBGC [56] is a newer deep learning software tool that uses a Bidirectional Long Short-Term Memory (BiLSTM) neural netword (RNN) and word2vec-like word embedding skip-gram neural network with three layers [56]. It uses an input layer of vectors of Pfam domains and genomic order, a layer of 128-dimensional hidden vectors, and the output layer of fully connected sigmoid functions, which is more sensitive (fewer false negatives) than ClusterFinder [56]. DeepBGC requires a large training data set for complex microbial communities.

In summary, the field of endophyte NP bioprospecting is ready for ‘ecometabolomic’ and ‘phylometabolomic’ deep learning, for example, using the H2O.ai deep learning framework [53]. Similar approaches are in use now in ecology [259] and there are increasingly more deep learning libraries for genomics, such as the recent python deep learning library, Janggu [260] which is compatible with other related python libraries; together, the goal will be to seamlessly integrate phylogenomic and hologenome predictions with interactome systems biology [261]. Arguably, the time to begin is now, given the rate of global plant habitat and biodiversity loss.

#### Deep learning for predicting the chemical structural diversity of endophytes

Machine learning and deep learning approaches have been developed for chemoinformatics, anti-cancer and antibiotic drug discovery, and metabolomics [262–265]. In particular, these approaches have been useful for organic chemical exploration [264], bioactivity prediction based on chemical structure and mapping BGC combinations to chemical groups. We suggest the next critical frontier will be to develop chemoinformatics and bioactivity-focused informatics that integrate with and inform bioprospecting. Specifically, research could focus on systematic computational learning approaches for predicting chemical structural diversity from endophytes based on integrated comparative metabolomics and chemical compound analysis, combined with biotic interaction network analysis, building a model of correlations between *in planta* biochemistry and plant microbioime ecology. Furthermore, these frameworks can be tailored according to specific goals. For example, alternative deep learning frameworks could focus on chemical novelty and dereplication, or specific bioactivities (e.g. antiviral vs. antifungal vs. anti-protozoan vs. antibacterial, or anticancer), or structures with the most complex synthesis such as (list chemical forms, bonds, or chirality groups).

Recent thinking on this topic is that it is critically important to avoid reductionism [266], because the power of these approaches is in their ability to address unknown interactions. Therefore, we suggest researchers should begin by training on encoded natural product chemical structural databases integrated with synthetic organic chemistry libraries and organismal metadata – particularly from habitat and metagenomic data. Because plants and plant-endophyte systems are targets for viral pathogens, they may hold promise for discovery of novel antiviral compounds, such as novel RNA-dependent RNA polymerase (RdRp) inhibitors, e.g. pyrazine family compounds related to pyrazinecarboxamides (e.g. favipiravir, currently in use as broad spectrum RdRp inhibitors against influenza and COVID-19). Similarly, plant-endophyte systems must defend against a wide range of fungal and bacterial pathogens and likely have evolved narrow-target antifungals and antibacterials. Animal-specific cytotoxic compounds are likely diverse in these systems, to combat a range of possible herbivore pests.

But what about uncultivatable endophytes, given that much research on endophyte NPs is motivated by the prospect that endophytes are easier to cultivate than plants [267, 268]? We argue that for uncultivatable endophytes, computational learning-based chemical structure prediction will be especially helpful for overcoming the need for isolation and synthesis, but also such approaches can narrow the search for targets for downstream experimental (and computational) unsilencing, as described below.

#### Deep learning for discovery of in planta unsilencing triggers – waking the sleeping giant

Hidden, or silenced biosynthetic capacities seem to be the rule, rather than the exception in plant microbiomes, as evidenced from bioinformatic identification of BGCs. This leads to a major research problem, that research has tried to overcome through co-cultivation, OSMAC experiments [28], heterologous expression experiments [232], high-throughput elicitor screening [47], transcription factor decoys [269], and *in planta* approaches [270]. Yet, to date, there has been little concerted effort to apply computational learning approaches to solve this problem. This would seem surprising, given that genome data mining methods exist to uncover a diversity of regulatory signaling processes, metabolic flux, metabolic pathway regulation, and holobiont metabolic interactions such as pathway complementation. Computational learning strategies could use training data that is already from high throughput elicitor or expression experiments, OSMAC arrays, combined with *in planta* or co-culture holometabolomic and holoregulomic data. One promising approach could be to incorporate trans-kingdom regulatory small RNA data, for example from miRNomics sequencing. Such approaches could be combined with unsilencing studies *in planta*, such as global effector studies on synthetic communities on gnotobiotic plants (SynCom), which have been used to analyze complex dynamics of effector secretion by pathogens and beneficials [270]. Finally, a major gap that could be addressed with deep learning is to investigate models of metabolic cooperation amongst endophytes and plants.

Thus, to increase the scope and throughput of BGC unsilencing experiments, we propose new in silico unsilencing pipelines that infuse comparative multi-omic analyses with deep learning. The result would be endophyte community-level ‘ecoregulomics’. With the blossoming world of software and bioinformatics approaches, this idea is arguably within reach.

## Conclusions

To meet the demand of the world’s emergent and resistant diseases caused by viruses (e.g. COVID-19), bacteria (e.g. tuberculosis), parasites (e.g. malaria), and other major illnesses and conditions, such as cancers, novel natural products will continue to be in demand. For plant microbiomes to fulfill their promise [20, 262] as a leading source of new antiviral, antibiotic, and anticancer drugs, higher throughput and computational approaches are needed. We have proposed integrating computational learning approaches (e.g. deep learning) into the pipeline for both predicting and validating novel endophyte metabolites. If implemented, such deep learning approaches could explore broader mysteries, for example, whether medicinal plant health benefits could derive from endophyte communities rather than plants, or whether cooperative biosynthetic pathways between host and microbe may be important in NP synthesis, for example, in Taxol. Endophyte-derived natural compounds may also be of value outside of medicine, for example, in buffering anthropogenic and climate effects or habitats and crops impacted by invasive pathogens [96, 271, 272]. All together, these points emphasize the need to conserve biodiversity with an enhanced focus on characterization and conservation of diverse endophyte-rich habitats.

## Supplementary Information

**Additional file 1.**

## Data Availability

Not applicable.

## Abbreviations

NP: Natural Product
SM: Secondary Metabolite
BGC: Biosynthetic Gene Cluster
